# Role of cytochrome phenotyping in patients with chronic noncancer pain with inadequate response to tramadol

**DOI:** 10.1097/PR9.0000000000001474

**Published:** 2026-07-17

**Authors:** Deborah Montmeat, Xavier Declèves, Alicja Puszkiel, Serge Perrot, Anne-Priscille Trouvin

**Affiliations:** a Pain Department, Cochin University Hospital, Paris Cité University, Assistance Publique Hôpitaux de Paris, Paris, France; b Inserm UMRS1144, Laboratory of Pharmacology and Toxicology, Cochin University Hospital, Paris Cité University, Assistance Publique Hôpitaux de Paris, Paris, France; c INSERM U987, Paris Cité University, Boulogne Billancourt, France; d Pain Department, Cochin University Hospital, Assistance Publique Hôpitaux de Paris, Paris, France

**Keywords:** Phenotypic drug cocktail, Cytochromes, Chronic pain, Personalised medicine, Opioid, Analgesics, CYP2D6

## Abstract

**Introduction:**

The opioid crisis is one of the most serious public health emergencies in the United States today. Opioid prescribing requires vigilance and reducing inappropriate opioid prescriptions is a key to decreasing opioid misuse. For that reason, we often focus solely on therapeutic indications or dosage while overlooking the polymorphisms of metabolism. Cytochromes P450 (CYP) are highly susceptible to interindividual and intraindividual variations and the analgesic activity of tramadol is dependent on the activity of CYP2D6.

**Objectives:**

To evaluate whether CYP phenotyping could identify metabolic abnormalities in patients with chronic non-cancer pain (CNCP) who experienced an inadequate response to tramadol and to assess the impact of phenotype-guided therapeutic adaptation on patient care.

**Methods:**

This exploratory study assessed CYP's activity using a phenotyping drug cocktail protocol adapted from the Geneva cocktail in patients suffering from CNCP who experienced an inadequate response to tramadol, either ineffectiveness or side effects leading to discontinuation of tramadol. Patients' drug therapy was subsequently tailored according to their cytochromes' activity, and its impact was evaluated one year later using patient-reported outcomes.

**Results:**

72.7% of patients who had side effects resulting in treatment discontinuation had slow metabolism for CYP2D6; 87.5% of patients who had experienced ineffectiveness with tramadol had slow metabolism for CYP2D6. The patients believed that this test led to a great improvement in their therapeutic management on average.

**Conclusion:**

An inadequate response to tramadol in a patient with CNCP could trigger consideration of metabolic phenotype exploration to facilitate personalised pain management and reduce unwarranted opioid prescription.

## Abstract 2

Supplemental Digital Content is Available in the Text.

Potential link was revealed between metabolism and inadequate response to tramadol. Cytochromes P450 phenotyping could be key to personalise pain management and reduce unwarranted opioid prescription.

## 1. Introduction

Opioid prescriptions require vigilance, and reducing inappropriate opioid prescriptions is a key factor in preventing opioid misuse.^35^ There is currently a major question regarding the usefulness of prescribing opioids to patients with chronic noncancer pain (CNCP), especially in light of the opioid crisis in the United States and its spread worldwide.^22^ Nevertheless, opioids have been cited in international recommendations for the management of chronic pain condition.^25^ Tramadol is a weak opioid administered as a racemic mixture and used to manage moderate to severe pain, including CNCP and acute pain.^12^ Among opioids, tramadol holds a distinct position as a second-line treatment for neuropathic pain and is, for example, given a “weak for” recommendation in the EULAR guidelines for managing fibromyalgia.^21^ Individualized analgesic treatment tailored to the indications of the patient, as well as the biopsychosocial context, is important to reduce the risk of abuse and misuse.

Tramadol consumption continues to increase in certain European countries and in some states of the United States.^3,29^ In France, tramadol is the most commonly prescribed opioid and the leading cause of death among medical users of analgesics, according to the DTA (“Décès Toxiques par Antalgique”) registry.^8,32^ Therefore, beyond its inclusion in management guidelines for various types of CNCP, tramadol was chosen as the inclusion criterion for this study.^28^ Moreover, tramadol is highly prone to adverse effects, and not all the toxicity mechanisms have been elucidated. Because this drug can exhibit multiple mechanisms of action—such as norepinephrine and serotonin reuptake inhibition alongside opioid receptor agonism—it becomes more challenging to distinguish the specific source of observed adverse effects.^4,15^

From a pharmacological standpoint, analgesic management is significantly influenced by cytochrome P450 2D6 (CYP2D6), as 3 commonly used opioids—oxycodone, codeine, and tramadol—require bioactivation through this enzyme. The CYP2D6 gene is highly polymorphic in the general population, leading to enzymes with varying functional activity. Depending on the specific genetic variant, CYP2D6 activity may be absent, reduced, normal, or increased. However, not all variants have been fully characterized, and in some cases, interpretation remains inconclusive.^23,33^ However, other factors can also affect CYP2D6 activity, including epigenetic regulation, food, plants, drugs, and liver impairment.^1^ This phenomenon is called phenoconversion; a patient with a given genotype exhibits enzyme activity of a different metabolizer group.^26^ Genotyping, by definition, only explores this gene. However, when the activity of CYP is evaluated with a dynamic test in vivo based on a drug cocktail approach, the response concerning the activity of CYP takes into account all parameters influencing CYP activity.^18^ This is known as phenotyping. The principle behind the drug cocktail cytochrome phenotyping tests is the administration of drugs to patients in small quantities. Each drug is metabolized explicitly by a single cytochrome. The drugs and metabolites were then assayed, and cytochrome activity was assessed. Because analgesics are typically used as adjuvant treatments, phenotyping may be more practical than genotyping, as their prescriptions can be more easily adjusted compared with more complex therapies such as anticancer agents.

The distribution frequency of these polymorphisms differs from one geographical origin to another. For example, Gaedigk et al.^14^ estimated that 5.44% of the European population lacks CYP2D6 activity, compared with 1.92% of the American population and 2.78% of the African population. Slow metabolizers accounted for 5.37% of Europeans, 2.77% of Americans, and 10.63% of Africans. To the best of our knowledge, no study has estimated the proportion of slow or fast metabolizers among patients using opioids, and opioid metabolism has rarely been assessed individually. This approach may help explain why some patients experience refractory pain despite high doses or, conversely, develop adverse effects at low doses—highlighting the potential need for medication adjustment.^13,27^

**Figure 1.**
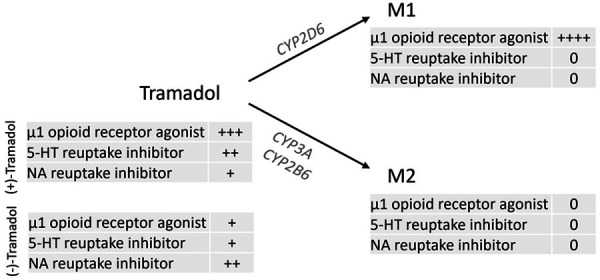
Metabolism of tramadol and activity of it and its metabolites. CYP2D6 mediated O-demethylation to metabolite M1 whereas CYP3A, with very minor participation of CYP2B6, catalyses the N-demethylation to metabolite M2. (+)-Tramadol acts mainly as a µ1 opioid receptor (OR) agonist and also acts to a lesser extent as a 5-hydroxytryptamine (5-HT) and Noradrenaline (NA) recapture inhibitor. (−)-Tramadol acts mainly as a NA reuptake inhibitor and also acts as a OR agonist and as a 5-HT reuptake inhibitor. M1 is responsible of the main opioid activity because it binds 300 times higher OR than tramadol. M2 has no activity. CYP, cytochrome P450.

Tramadol, our molecule of interest, has both direct activity and partial pharmacological dependence on cytochrome P450 enzymes.^12^ CYP2D6 is primarily responsible for O-demethylation, leading to the formation of the active metabolite M1, while CYP3A—along with minor contributions from CYP2B6—catalyzes N-demethylation to form M2. Tramadol exerts its analgesic effect mainly through μ1 opioid receptor agonism—primarily through M1, which has approximately 300-fold greater receptor affinity than the parent compound—as well as through monoamine reuptake inhibition (Fig. 1). Consequently, CYP2D6 activity significantly influences tramadol's overall analgesic efficacy.^11,16^

This study investigated the activities of CYP2D6 and CYP3A in a cohort of 41 patients with CNCP who had an inadequate response to tramadol, either because tramadol was ineffective at therapeutic doses or because it resulted in moderate-to-severe side effects that required treatment discontinuation.

The primary aim of this exploratory study was to determine the proportion of patients whose inadequate response could be attributed to a specific metabolic profile. We investigated the patient's CYP phenotype using a phenotyping cocktail. In the second phase, we re-evaluated the impact of this personalized analgesic prescription based on phenotyping during follow-up hospital appointments.

The long-term objective of this exploratory study was to assess the significance and feasibility of CYP phenotyping in CNCP and determine whether the routine use of CYP phenotyping could improve treatment efficacy and tolerability.

## 2. Methods

### 2.1. Study design and ethics statement

This monocentric prospective cohort study was performed at Cochin University Hospital in France. This study was approved by the French Ethics Committee of the *Comité de Protection des Personnes Sud Méditerranée* (Agreement No. ID-RCB-2017-A00685-48). Written informed consent was obtained from all patients participating in the study.

### 2.2. Patients

Patients were enrolled between November 2021 and November 2022. Inclusion criteria were as follows: adult patients (18 years and older) who suffer from CNCP and have experienced ineffectiveness or poor tolerance to tramadol within the past 5 years, the poor tolerance having to be significant enough to have led to discontinuation of treatment. Inclusion occurred during the first consultation in the Pain Management Department (PMD), and a physician determined the inclusion criteria were during an oral interview. Effectiveness and adverse effects were evaluated on an 11-point Likert scale ranging from 0 to 10. Zero represents absolute ineffectiveness and the total absence of adverse effects. Ten represents the optimal efficacy and strongest side effects.

Tramadol-naïve patients and those with good tolerability and adequate efficacy were excluded based on the clinical context.

This was an exploratory study of standard care; therefore, no well-tolerated control group was established.

### 2.3. Phenotyping

Patients were admitted to the study session at the PMD after an 8-hour fasting period. Patients who took medication in the morning delayed their intake until their blood samples were collected. A phenotyping cocktail was orally administered to patients. It was adapted from the phenotyping Geneva cocktail^7^ according to the drug dosages available in France^9^: caffeine 50 mg, bupropion 150 mg, flurbiprofen 50 mg, omeprazole 10 mg, dextromethorphan 10 mg, midazolam 1 mg. These components explore the activities of CYP1A2, CYP2B6, CYP2C9, CYP2C19, CYP2D6, and CYP3A, respectively (Table 1).

**Table 1:** Formula of drug cocktail adapted from Geneva cocktail with dosages available in France.

Probe	Dosage (mg)	Drug formulation	Enzymatic target	Measured metabolite	Ratio calculation
Dextromethorphan	10	Syrup, 25 mg/10 mL	CYP2D6	Dextrorphan	[Dextrorphan]/[Dextromethorphan]
Midazolam	1	Injectable solution, 5 mg/5 mL	CYP3A	1-hydroxymidazolam	[1-hydroxymidazolam]/[Midazolam]
Flurbiprofen	50	Coated tablet	CYP2C9	4-hydroxyflurbiprofen	[4-hydroxy flurbiprofen]/[Flurbiprofen]
Caffeine	50	Injectable/oral solution, 50 mg/2 mL	CYP1A2	paraxanthine	[paraxanthine]/[Caffeine]
Bupropion	150	Film-coated extended-release tablet	CYP2B6	4-hydroxybupropion	[4-hydroxybupropion]/[Bupropion]
Omeprazole	10	Gastro-resistant capsule	CYP2C19	5-hydroxyomeprazole	[5-hydroxyomeprazole]/[Omeprazole]

CYP, cytochrome P450.

**Figure 2.**
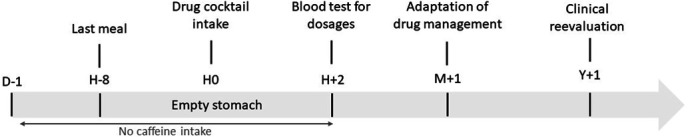
Phenotyping protocol. D, day; H, hour; M, month; Y, year.

Because our primary focus was on the tramadol response in patients with CNCP, we only present the activities of the CYP enzymes involved in the metabolism of CYP2D6 and CYP3A. Two hours after the cocktail was administered, blood samples were collected for pharmacological assays to evaluate the activity of the CYP of interest (Fig. 2). The administered drugs and their metabolites were quantified in plasma using a previously described High-performance liquid chromatography - tandem mass spectrometry (HPLC-MS/MS) method.^5,7^ The CYP activity was assessed using specific metabolite/probe concentration ratios (metabolic ratios [MRs]). The MRs yielded 3 groups of patients: those with slow metabolism (SM) (including both poor metabolism [PM] and intermediate metabolism [IM] for CYP2D6 and PM for CYP3A), those with normal metabolism (NM), and those with ultra-rapid metabolism (UM).^18,20^

### 2.4. Demographic characteristics

For each patient, we recorded demographic data, including sex, age, type of pain disorder, use of grapefruit and St. John's wort, and current medications. Based on their summary of product characteristics, these medications were classified as CYP inhibitors or inducers.

### 2.5. Clinical evaluation

Patients were asked to evaluate the CYP phenotyping test, including the difficulty of performing the test, the level of stress and constraints it caused, and to report any side effects they may have experienced during the test. Moreover, during their follow-up hospital appointment, they were asked to evaluate their pain management, implemented according to the phenotyping results, one year after undergoing the test, between November 2022 and November 2023. Patients rated their health improvement regarding pain management using the Patient Global Impression of Change (PGIC) score, which used a 7-point scale: 1. Very much improved, 2. Much improved, 3. Minimally improved, 4. No change, 5. Worse, 6. Much worse, 7. Very much worse.

During the one-year follow-up, patients were monitored at the PMD, and their management was adapted based on the results of cytochrome phenotyping. In other words, patients with a slow CYP2D6 phenotype were not reintroduced to the analgesic prodrugs. In addition, it was assumed that patients who experienced adverse reactions to tramadol and had a normal CYP2D6 phenotype likely had a general intolerance to opioids; therefore, no opioids were reintroduced. Depending on the etiology of the pain, several management options were undertaken, classified as follows: (1) topical treatment (capsaicin 8% dermal patch or topical lidocaine or topical nonsteroidal anti-inflammatory), (2) switch to an opioid that does not require CYP2D6 activation (opium powder), (3) switch to a nonopioid analgesic, (4) transcutaneous electrical nerve stimulation (TENS), (5) discontinuation of oral treatments, and/or (6) nonpharmacologic treatment (kinesitherapy, balneotherapy, exercise, weight loss).

### 2.6. Data and statistical analysis

Sample size calculation: This exploratory study did not calculate the sample size because there were no a priori hypotheses. It aimed to understand the prevalence of a particular phenotype in patients with inadequate response to tramadol and explore the potential positioning of phenotyping as a routine care test for patients with CNCP.

Categorical and continuous variables are described using frequency tables (n, %) and mean with standard deviation (SD).

Statistical analyses were performed on R software, version 4.2.2.

## 3. Results

### 3.1. Population characteristics

Between November 2021 and November 2022, 41 patients were admitted for the study and underwent phenotyping. The participants' ages ranged from 22 to 82 years (49.2 ± 16.2, mean ± SD). There were 8 men (19.5%) and 33 women (80.5%). None of the participants had consumed grapefruit or St. John's wort during the 2 weeks before the test. Nineteen patients (46.3%) had nociceptive pain, 13 (31.7%) had neuropathic pain, and 9 (22%) had nociplastic pain (Table 2). Full details of the clinical data—the specific clinical context and the drugs taken by patients that influenced the activity of CYP— are summarized in the Supplementary Data (Table S1, http://links.lww.com/PR9/A435).

**Table 2:** Characteristics of patients and type of inadequate response to tramadol.

Demographic characteristics of patients	
Sex (male/female), No. (%)	8 (19.5)/33 (80.5)
Age, mean ± SD	49.2 ± 16.2
Type of pain	
Neuropathic pain, No. (%)	13 (31.7)
Nociplastic pain, No. (%)	9 (22)
Nociceptive pain, No. (%)	19 (46.3)

Type of inadequate response of tramadol	
Group A: side effects resulting in treatment discontinuation, No. (%)	
Evaluation of side effects in 11 points	33 (80.5)
Likert scale from 0 to 10, mean ± SD	8.2 ± 1.4
Group B: ineffectiveness, No. (%)	
Evaluation of effectiveness in 11 points	8 (19.5)
Likert scale from 0 to 10, mean ± SD	1 ± 1.9

Thirty-three participants (80.5%) experienced moderate-to-severe side effects, leading to the discontinuation of tramadol (group A), while 8 participants (19.5%) experienced tramadol ineffectiveness (group B). In group A, the severity of side effects was rated on average at 8.2 ± 1.4 on an 11-point Likert scale. In group B, the effectiveness was rated at 1 ± 1.9 (Table 2). There was no overlap between the 2 groups, and no participants reported inefficacy or side effects.

### 3.2. Pain management and clinical evaluation

Of the 41 patients enrolled, 33 remained in follow-up at the PMD and were available for reevaluation. Thirty-two patients reported that the phenotyping test was well-tolerated, with no adverse effects. One patient experienced mild nausea but still rated their global impression of change (PGIC) as 2.

Notably, 2 cases stood out. Both patients had been on high-dose tramadol therapy (>300 mg/d) for over 5 years. Phenotyping revealed that they have slow metabolism for CYP2D6 (SM-CYP2D6). After this finding, both were able to discontinue tramadol and transition rapidly to opium powder. They reported significant improvements in overall condition and better pain control with opium compared with tramadol.

Full details of pain management in patients are summarized in the Supplementary Data (Table S2, http://links.lww.com/PR9/A435).

### 3.3. Group A: patients who experienced side effects leading to the discontinuation of tramadol

#### 3.3.1. Cytochrome P450 phenotype distribution

Of the 33 patients evaluated, 24 (73%) exhibited a SM-CYP2D6, while 27% had normal metabolism for CYP2D6 (NM-CYP2D6). No patients were identified as ultra-rapid metabolizers (UM-CYP2D6). Patients with the SM-CYP2D6 phenotype rated tramadol effectiveness at an average of 1.6 ± 2.9 of 10, whereas those with the NM-CYP2D6 phenotype rated it higher, at 4.1 ± 4.5 of 10. In addition, within this cohort, 90% of patients carried the NM-CYP3A phenotype (Table 3).

**Table 3:** Distribution of measured phenotypes for CYP2D6 and CYP3A and evaluation of efficacy and side effects in 11-point Likert scale from 0 to 10 according to type of inadequate response and type of phenotype of CYP2D6.

		SM		NM	UM
		PM	IM		
CYP2D6	All patients, No. (%)	3 (7)	28 (68)	10 (24)	0
	Group A, No. (%)	3 (9)	21 (64)	9 (27)	0
	Evaluation of efficacy, mean ± SD	1.6 ± 2.9		4.1 ± 4.5	
	Evaluation of side effects, mean ± SD	8.2 ± 1.4		8.2 ± 1.6	
	Group B, No. (%)	0	7 (88)	1 (12)	0
	Evaluation of efficacy, mean ± SD	1.1 ± 2.0		0	
	Evaluation of side effects, mean ± SD	0.3 ± 0.8		0	
CYP3A	All patients, No. (%)	4 (10)	—	36 (90)	0
	Group A, No. (%)	3 (9)	—	29 (90)	0
	Group B, No. (%)	1 (12)	—	7 (88)	0

CYP, Cytochrome P450; IM, intermediate metabolism; NM, normal metabolism; PM, poor metabolism; SM, slow metabolism; UM, ultra-rapid metabolism.

#### 3.3.2. Side effects

Among SM-CYP2D6 patients, commonly reported side effects included nausea and vomiting (46%), confusion (38%), dizziness (38%), and drowsiness (21%). Other symptoms included headache (13%), sleep disturbances (8%), and dry mouth (4%). By contrast, NM-CYP2D6 patients predominantly experienced central nervous system side effects: confusion (56%), dizziness (56%), and drowsiness (33%). Gastrointestinal symptoms were also present in this group, with nausea or vomiting reported by 33% and dry mouth by 22%. A complete summary of adverse events is available in Supplementary Table S3, http://links.lww.com/PR9/A435. Some of these side effects could be due to serotonin excess or an high sensitivity to the limited opioid receptor effect.

#### 3.3.3. Management of patients with neuropathic pain

Among the 9 patients who experienced poor tolerability to tramadol, all but 2 had the SM-CYP2D6 phenotype. Management strategies for NM-CYP2D6 patients included local treatment with a capsaicin 8% dermal patch and discontinuation of all oral medications, yielding PGIC scores of 1 and 4. In the SM-CYP2D6 group, one patient with small fiber neuropathy received only nonpharmacological interventions (eg, kinesiotherapy and gradual physical activity), with a PGIC score of 2. The remaining 6 patients received topical treatments, opium powder, or both in combination, reporting a mean PGIC score of 1.3.

#### 3.3.4. Management of patients with nociceptive pain

Of the 10 patients with nociceptive pain and poor tramadol tolerability, 9 had PGIC scores of 1 or 2. Two of these patients had the NM-CYP2D6 phenotype and were switched to the nonopioid analgesic paracetamol. Patients with the SM-CYP2D6 phenotype were either managed with comprehensive care, discontinued oral medications, or transitioned to opium powder. Only one SM-CYP2D6 patient reported a PGIC score of 4.

#### 3.3.5. Management of patients with nociplastic pain

Among the 4 patients who discontinued tramadol due to adverse effects, 3 ceased all oral pharmacological treatments and were managed with nonpharmacological interventions, including kinesiotherapy and progressive physical activity. These patients reported a PGIC score of 2. The remaining patient, who had a history of moderate to severe pain (≥5/10 during acute episodes), was treated with a topical nonsteroidal anti-inflammatory drug and maintained a rescue analgesic on an as-needed basis (less than once per week). Given its SM-CYP2D6 phenotype, opium powder was prescribed, resulting in a PGIC score of 1.

#### 3.3.6. Summary of global patient-reported outcomes

Patients with SM-CYP2D6 reported a mean PGIC score of 1.4 ± 0.8, while those with NM-CYP2D6 reported a mean score of 2.4 ± 1.3.

### 3.4. Group B: patients with tramadol ineffectiveness

#### 3.4.1. Cytochrome P450 phenotype distribution

Among the 8 patients who discontinued tramadol due to lack of efficacy:

(1) Seven (88%) exhibited the SM-CYP2D6 phenotype.
(2) 88% also carried the NM-CYP3A phenotype.
(3) None were classified as UM for either CYP2D6 or CYP3A (Table 3).

#### 3.4.2. Management of patients with neuropathic pain

All patients with neuropathic pain in this group had the SM-CYP2D6 phenotype. Treatment consisted of topical application of a capsaicin 8% transdermal patch and replacement of the rescue analgesic with opium powder. Reported PGIC scores were 1 and 2.

#### 3.4.3. Management of patients with nociceptive pain

All 4 patients with nociceptive pain and tramadol ineffectiveness were identified as SM-CYP2D6. Two patients discontinued oral treatments and were managed exclusively with nonpharmacological therapies. The remaining 2 patients were transitioned to opium powder. The mean PGIC score for this subgroup was 1.25.

#### 3.4.4. Management of patient with nociplastic pain

One patient with nociplastic pain and tramadol inefficacy was found to have the NM-CYP2D6 phenotype. Because treatment failure could not be attributed to metabolic factors, oral analgesics were discontinued. The patient was treated with nonpharmacological modalities and reported a PGIC score of 4.

#### 3.4.5. Summary of global patient-reported outcomes

Final PGIC scores were as follows:

(1) SM-CYP2D6: Mean PGIC = 1.3 ± 0.5
(2) NM-CYP2D6: Mean PGIC = 4.0

Although NM-CYP2D6 patients reported slightly higher PGIC scores than those with SM-CYP2D6, this difference may reflect the limited response to tramadol expected in slow metabolizers, as identified through CYP phenotyping.

## 4. Discussion

### 4.1. Correlation between phenotyping and inadequate response to tramadol

First, most patients who did not experience analgesic effects with tramadol harbored SM-CYP2D6 (87.5%), but no association with the CYP3A phenotype was found. These results agree with numerous previous studies on the subject, including those conducted as early as 1996.^30^ Because M1 is the metabolite responsible for most of the analgesic activity, individuals who do not form M1 will not experience an adequate analgesic effect.

However, results from patients who demonstrated poor tolerability to tramadol are controversial. Most patients had SM-CYP2D6 (72.7%), and no association was found with the CYP3A phenotype. The literature often states that patients with SM-CYP2D6 are less prone to adverse reactions. In our study, the side effects presented by this population were mostly gastrointestinal symptoms, such as nausea or vomiting, and changes in mental status, such as confusion or dizziness. These symptoms may reflect an excess of serotonin in the 5-hydroxytryptamine (5-HT) receptors. In 2015, Beakley et al.^4^ suggested that the risk of serotonin syndrome with tramadol in patients with SM-CYP2D6 could exist even in the absence of risk factors, such as the coadministration of drugs. However, these symptoms, as well as serotonin syndrome, have been identified when tramadol is coadministered with CYP2D6 inhibitors such as fluoxetine, paroxetine, or mirtazapine.^17,19^ For patients with SM-CYP2D6, it is possible that, while they accumulate tramadol, they do not form M1, making them more susceptible to developing side effects related to excess serotonin levels. It is also important to highlight that the patients in our cohort had CNCP, a particularly vulnerable population to the nocebo effect. As noted in a meta-analysis by Mitsikostas et al.,^24^ 67.2% of patients with fibromyalgia who received a placebo reported at least one side effect, and 9.5% had to discontinue placebo treatment due to intolerance. It remains possible that the side effects reported by patients were influenced by a nocebo effect, independent of CYP metabolic activity. Moreover, another potential confounding factor—genetic variability in the opioid receptor gene OPRM1 and in catechol-O-methyltransferase, both of which can affect opioid response—is difficult to evaluate due to the absence of relevant clinical data guiding pharmacological management.^23^

### 4.2. Phenotyping and tailored pain management

This exploratory study aimed to investigate the phenotypic activity of CYP2D6 and CYP3A in patients with CNCP who showed an inadequate response to tramadol, weak efficacy, or poor tolerability. Patient inclusion criteria were based on tolerance to tramadol and not on the type of CNCP; therefore, patients with a wide variety of CNCP could be evaluated. We analyzed the CYP phenotypes in a cohort of 41 patients and found a significant association between clinical response and CYP profile. The assessment of CYP activity was based on a widely recognized drug cocktail approach described in the literature,^6,7,18,20^ and pain management was adjusted according to the identified CYP activities.

Our findings strongly suggest that patients with CNCP who exhibit an inadequate response to tramadol are predominantly characterized by a specific CYP2D6 phenotype, with 76% identified as slow metabolizers. In the context of pain management, phenotyping seems more appropriate than genotyping, as analgesics are typically used as supportive, rather than curative, treatments. Altering long-term therapies prescribed for chronic comorbid conditions solely based on CYP status would be unwarranted, particularly given the availability of alternative analgesics that are not metabolized by cytochrome P450 enzymes.

While CYP2D6 plays a critical role in the metabolism of several opioids—including the commonly used weak opioid codeine^10^—other weak opioids, such as tapentadol and opium powder-based formulations, do not rely on CYP2D6 for activation. The availability of these alternatives, however, varies by country. Tapentadol, for example, exerts its analgesic effects through a dual mechanism—μ-opioid receptor agonism and inhibition of norepinephrine reuptake—but its use could not be assessed in this study due to its unavailability in France.^34^ Importantly, neither tapentadol nor opium powder requires CYP2D6 for analgesic efficacy.^2^

### 4.3. Limitations

One limitation of this study relates to the use of bupropion in the phenotyping cocktail, as it is known to interact with CYP enzymes. While bupropion has demonstrated inhibitory effects on CYP2D6 activity when administered twice daily and at steady-state concentrations, there is no evidence that a single-dose administration—such as that used in this protocol—produces a similar effect.^31^ The developers of the Geneva cocktail, from which our protocol was adapted, demonstrated that the combination of drugs used does not result in mutual pharmacokinetic interactions.^6^

Another limitation is the potential for recall bias, as some patients were included based on self-reported inadequate responses to tramadol received in the past. This retrospective element may affect the accuracy of patient-reported outcomes.

In conclusion, an inadequate response to tramadol in patients with CNCP should prompt the exploration of cytochrome phenotypes, as this can significantly influence pain management strategies. Cytochrome P450 phenotyping has proven to be a valuable tool for physicians to tailor their treatment plans. Specifically, patients with SM-CYP2D6 who require opioid-based pain management can be treated with either tapentadol or opium powder-based medications, depending on their availability in different countries.

## Disclosures

The authors have no conflict of interest to declare.

## Supplementary Material

## Supplemental digital content

Supplemental digital content associated with this article can be found online at http://links.lww.com/PR9/A435.
