# Supplementary material for: Role of cytochrome phenotyping in patients with chronic noncancer pain with inadequate response to tramadol
Source: Pain Rep. 2026 Jul 17;11(4):e1474. doi: 10.1097/PR9.0000000000001474 (PMC13384671; doi:10.1097/PR9.0000000000001474)
Supplement: Supplementary file 1 [file painreports-11-e1474-s001.pdf]

Table S1. Details of the patients' clinical data

Patient number	Age (years)	Gender	Tobacco consuption	Grapefruit consuption	St. John's wort consuption	Type of CNCP	Type of inadequate response to tramadol	Inhibitor or inducer of CYP2D6 among current treatments	Inhibitor or inducer of CYP3A4 among current treatments	Phenotype of CYP2D6	Phenotype of CYP3A4
1	44	F	No	No	No	Neuropathic pain	Not tolerated	No	No	NM	NM
2	62	F	No	No	No	Neuropathic pain	Inefficiency	No	No	IM	NM
3	72	F	No	No	No	Nociplastic pain	Not tolerated	No	No	IM	NM
4	79	F	No	No	No	Nociceptive pain	Not tolerated	Yes, inhibitor: venlafaxine	No	IM	NM
5	37	F	No	No	No	Neuropathic pain	Not tolerated	No	No	IM	NM
6	42	M	No	No	No	Neuropathic pain	Not tolerated	No	Yes, inhibitors: tacrolimus, doxycycline	IM	NM
7	58	F	No	No	No	Nociceptive pain	Not tolerated	Yes, inhibitor: metoclopramide	No	NM	NM
8	79	F	No	No	No	Neuropathic pain	Not tolerated	No	No	IM	NM
9	24	F	No	No	No	Neuropathic pain	Not tolerated	No	No	NM	NM
10	38	M	No	No	No	Nociplastic pain	Not tolerated	Yes, inhibitor: duloxetine	No	IM	PM
11	23	F	No	No	No	Nociceptive pain	Not tolerated	Yes, inhibitor: venlafaxine	No	IM	NM
12	26	F	No	No	No	Neuropathic pain	Not tolerated	Yes, inhibitor: fluoxetine	No	PM	NM
13	49	F	No	No	No	Nociceptive pain	Not tolerated	No	No	IM	PM
14	67	M	No	No	No	Nociceptive pain	Inefficiency	No	No	IM	PM
15	53	M	No	No	No	Nociplastic pain	Not tolerated	No	No	NM	NM
16	58	F	No	No	No	Nociplastic pain	Not tolerated	No	No	NM	NM
17	53	F	No	No	No	Nociplastic pain	Not tolerated	Yes, inhibitor: duloxetine	No	IM	NM
18	55	F	No	No	No	Neuropathic pain	Not tolerated	Yes, inhibitor: duloxetine	No	IM	NM
19	51	F	No	No	No	Nociceptive pain	Not tolerated	Yes, inhibitor: paroxetine	No	NM	NM
20	24	F	Yes	No	No	Nociplastic pain	Not tolerated	No	No	NM	NM
21	82	F	No	No	No	Nociceptive pain	Not tolerated	No	No	IM	NA
22	43	F	No	No	No	Nociceptive pain	Not tolerated	No	No	IM	NM
23	65	F	No	No	No	Nociceptive pain	Not tolerated	No	No	IM	NM
24	35	F	No	No	No	Nociceptive pain	Not tolerated	No	No	NM	NM

Patient number	Age (years)	Gender	Tobacco consuption	Grapefruit consuption	St. John's wort consuption	Type of CNCP	Type of inadequate response to tramadol	Inhibitor or inducer of CYP2D6 among current treatments	Inhibitor or inducer of CYP3A4 among current treatments	Phenotype of CYP2D6	Phenotype of CYP3A4
25	22	M	No	No	No	Nociplastic pain	Not tolerated	No	No	IM	NM
26	52	F	No	No	No	Nociceptive pain	Inefficiency	No	No	IM	NM
27	43	M	No	No	No	Nociceptive pain	Inefficiency	Yes, inhibitor: venlafaxine	No	IM	NM
28	49	M	No	No	No	Neuropathic pain	Inefficiency	No	No	IM	NM
29	64	F	No	No	No	Nociceptive pain	Inefficiency	No	No	IM	NM
30	56	F	No	No	No	Nociplastic pain	Inefficiency	No	No	NM	NM
31	34	F	No	No	No	Nociplastic pain	Not tolerated	No	No	IM	NM
32	62	F	No	No	No	Nociceptive pain	Inefficiency	No	No	IM	NM
33	42	F	No	No	No	Nociceptive pain	Not tolerated	No	No	IM	NM
34	73	F	No	No	No	Neuropathic pain	Not tolerated	Yes, inhibitor: paroxetine	No	PM	NM
35	28	F	No	No	No	Nociceptive pain	Not tolerated	No	No	NM	NM
36	49	F	No	No	No	Neuropathic pain	Not tolerated	No	No	IM	NM
37	60	F	No	No	No	Neuropathic pain	Not tolerated	No	No	IM	NM
38	41	F	No	No	No	Nociceptive pain	Not tolerated	No	No	IM	NM
39	30	F	No	No	No	Neuropathic pain	Not tolerated	No	No	IM	NM
40	46	F	No	No	No	Nociceptive pain	Not tolerated	No	No	PM	NM
41	46	M	Yes	No	No	Nociceptive pain	Not tolerated	No	No	IM	PM

Table S2. All side events reported by patients in group A and group B

	Group A		Group B	
	SM for CYP2D6	NM for CYP2D6	SM for CYP2D6	NM for CYP2D6
**Gastrointestinal disorder**				
nausea/vomiting	11	3	-	-
constipation	4	-	-	-
gastric pain	2	-	-	-
oral dryness	1	2	-	-
diarrhea	1	-	-	-
**Central nervous system disorder**				
confusing	9	5	-	-
dizziness	9	5	1	-
drowsiness	5	3	-	-
sleeping disorders	2	1	-	-
headache	3	-	-	-
tremors	2	-	-	-
fall	2	-	-	-
lack of appetite	1	-	-	-
hallucination	-	1	-	-
euphoria	-	1	-	-
**Cardiovascular disorder**				
tachycardia	1	-	-	-
chest tightness	-	1	-	-
**Others**				
sweatings	2	-	-	-
skin rashes	2	-	-	-
tinglings	1	-	-	-

Table S3. Detail of changes in therapeutic management classified into 6 categories

local treatment (capsaicin 8% dermal patch or topical lidocaine or topical non-steroid anti-inflammatory), switch to an opioid that does not require an activation by CYP2D6 (opium powder), switch to a non-opioid analgesic (paracetamol), transcutaneous electrical nerve stimulation (TENS), discontinuation of oral treatments, and/or non-pharmacological treatment (kinesitherapy, balneotherapy, sport, weight loss). One patient may fit into the criteria for several managements presented.

		Local treatment	Switch to an opioid that does not require activation by CYP2D6	Switch to a non-opioid analgesic	TENS	Oral treatments discontinuation	Non-pharmacological treatment: kinesitherapy, balneotherapy, sport, weight loss	Not evaluable because of major change in clinical context
**Group A**	**SM for CYP2D6**	5	7	1	1	2	7	2
	**NM for CYP2D6**	1	-	2	-	-	1	-
**Group B**	**SM for CYP2D6**	2	4		1	1	1	-
	**NM for CYP2D6**	-	-	-	-	-	1	-
